# How Does an Observational Assessment Adapted for Online Delivery Perform Compared to an In-Person Assessment? Learning From the National Autism Service for Adults

**DOI:** 10.1192/bjo.2022.225

**Published:** 2022-06-20

**Authors:** Charlotte Blackmore, Alexandra Nolan, Vladimira Stoencheva, Natalie Greenwood, Natasha Liu-Thwaites, Stefanos Maltezos, Grainne McAlonan

**Affiliations:** 1 South London and Maudsley NHS Foundation Trust, London, United Kingdom; 2 King's College London, London, United Kingdom

## Abstract

**Aims:**

The National Autism Service for Adults receives over 600 referrals annually and with an extensive waitlist, COVID-19 restrictions on in-person assessments were a challenge for service delivery. We aimed to adapt the Autism Diagnostic Observation Schedule (ADOS) for online delivery and investigate whether it is comparable to the in-person ADOS in predicting Autism Spectrum Disorder (ASD) diagnostic outcome. We also aimed to obtain qualitative feedback from service users and clinicians regarding experiences of the online ADOS.

**Methods:**

A working group of staff who administer ADOS and representatives from psychiatry, psychology and management reached consensus that an online version of ADOS module 4 was feasible based on experience that a lot information required for coding is obtained verbally and some tasks were adaptable for online delivery. After the pilot, it was agreed all algorithm items could be coded except ‘unusual eye-contact’. Subsequently, 163 service users attended an online ADOS between August 2020 and February 2021. A matched-comparison group consisted of 198 service users seen for an in-person ADOS between May 2014 and February 2020. Algorithm scores were recorded and ASD diagnosis was made by a trained clinician. Qualitative feedback regarding the online ADOS was collected from 46 service users and 11 clinicians.

**Results:**

The working group agreed the online and in-person ADOS were closely matched regarding administration and coding. Mean scores for service users who received an ASD diagnosis were comparable for the online and in-person ADOS groups (7 and 8 respectively). This was also shown for those who were not diagnosed with ASD (3 and 4 respectively). A two-sample t-test showed no significant difference in total scores between the online and in-person ADOS (p = 0.38). Qualitative feedback suggested good service user and clinician satisfaction; only 27% of service users indicated they would have preferred an in-person assessment; 88% of clinicians reported there were gains from offering an online alternative. Although the online and in-person ADOS perform similarly, clinicians reported relying more on qualitative reports over scores from the online version to inform diagnostic decision.

**Conclusion:**

To our knowledge, this is the first study to examine using an online ADOS within an adult diagnostic service. Due to its comparable performance, the online-ADOS is a viable alternative option for service delivery when in-person assessments are not possible. As this clinic group has high rates of comorbid mental health difficulties, the applicability of online assessments could generalise to other services and have an impact beyond the pandemic.

